# Trends and off-label utilization of antipsychotics in children and adolescents from 2016 to 2021 in China: a real-world study

**DOI:** 10.1186/s13034-024-00766-4

**Published:** 2024-06-21

**Authors:** Wang Zhaojian, Jiang Meizhu, Hong Jun, Guo Shanshan, Huo Jiping, Zhao Zhigang, Gong Ying, Li Cao

**Affiliations:** 1 Department of Pharmacy, Beijing Tiantan Hospital, Capital Medical University, 119 Nan Si Huan Xi Lu, Fengtai District, 100050 Beijing, China; 2 Department of Pharmacy, Beijing Shijitan Hospital, Capital Medical University, Beijing, China; 3 Department of Pharmacy, Beijing Chao-Yang Hospital, Capital Medical University, Beijing, China; 4 Department of Clinical Pharmacology, School of Pharmaceutical Sciences, Capital Medical University, Beijing, China; 5 Department of Pharmacy, Dongfang Hospital, Beijing University of Chinese Medicine, No. 6, Phase 1, Fangxingyuan, Fangzhuang, Fengtai District, Beijing, China

**Keywords:** Antipsychotics, Trends, Off-label, Children and adolescents, Pediatrics

## Abstract

**Background:**

Global antipsychotic usage, including off-label prescriptions, has increased in recent decades. However, trends in China, particularly for children and adolescents, remain unclear. This study explored these trends from 2016 to 2021 and identified factors associated with off-label prescriptions.

**Methods:**

In this retrospective study, we analyzed on-label and off-label prescriptions based on drug information approved by the China National Medical Products Administration. To identify factors associated with off-label prescriptions, we conducted multivariate logistic regression analysis.

**Results:**

Our study included 48,258 antipsychotic prescriptions, 52.4% (25,295) of which were prescriptions for males. Of these, 61.7% (29,813) were off-label. Over time, the number of antipsychotics and the percentage of off-label prescriptions for children and adolescents overall increased from 2016 to 2021. The use of atypical antipsychotics increased, whereas that of typical antipsychotics decreased. For off-label usage, all of the factors in our study were associated with off-label usage, including age, sex, year, region, department, reimbursement, antipsychotic type, drug expense, number of polypharmacy and diagnoses. Additionally, tiapride (15.8%) and aripiprazole (18.6%) were the most common typical and atypical antipsychotics, respectively. For pediatric diseases, common diagnoses included mood or affective disorders (31.7%) and behavioral and emotional disorders, with onset usually occurring in childhood and adolescence (29.1%). Furthermore, a depressive state was the most common diagnosis for which antipsychotic polypharmacy was used for treatment.

**Conclusion:**

In this retrospective study, off-label antipsychotic prescriptions were common, with trends generally increasing among children and adolescents from 2016 to 2021. However, there is a lack of evidence supporting off-label usage, thus emphasizing the need for studies on the efficacy and safety of these treatments.

**Supplementary Information:**

The online version contains supplementary material available at 10.1186/s13034-024-00766-4.

**Key points**

1. From 2016 to 2021, off-label antipsychotic use in pediatric patients increased.
2. The use of atypical antipsychotics increased, whereas the use of typical antipsychotics decreased, which was consistent for both off-label atypical and typical antipsychotics.
3. Given the relatively high prevalence of off-label usage and potential inappropriate use of antipsychotic polypharmacy, there is a need for support from evidence-based research.

## Introduction

The global prevalence of psychosis is estimated to be 50 per 100,000 individuals, with schizophrenia more commonly occurring in approximately 15 per 100,000 individuals [1, 2]. Key features of psychosis include hallucinations, delusions, and cognitive impairments, which greatly impact daily life and require standardized treatment. Although psychosis in pediatric patients is rare compared to that in adults, the number of affected patients remains substantial, thus raising ongoing concerns [3–5].

Based on guidelines for the treatment of psychosis, both psychological intervention and pharmacotherapy can be utilized as early treatments for patients with psychosis [6, 7]. Meanwhile, these guidelines have indicated that early use of antipsychotics (APs) could reduce or delay the onset of psychosis. According to the underlying mechanism, Aps are categorized into typical and atypical antipsychotics, also known as first-generation antipsychotics (FGAs) and second-generation antipsychotics (SGAs), for the treatment of schizophrenia, bipolar disorders and other behavioral disorders [8, 9]. Compared to FGAs, SGAs have fewer neurological adverse reactions but are associated with a greater risk of metabolic adverse events [10, 11]. As a vulnerable population, children and adolescents are seldom included in clinical trials [12–14]. Moreover, restrictive marketing authorizations may also be responsible for the higher off-label rate in children and adolescents [15, 16]. This has caused widespread concern about the efficacy and safety of antipsychotics in pediatric patients because psychiatric adverse events are more likely to be observed in pediatric patients [17, 18].

In recent years, there has been a concern about the use of antipsychotics, including off-label prescriptions, in children and adolescents worldwide [19–23]. Similar to adults, drug safety monitoring measures (such as laboratory tests) are recommended for children and adolescents [24–26] to address the higher rate of adverse events in pediatric patients early in life [27]. Although off-label prescriptions are more prevalent in pediatric patients, studies have indicated that there is no significant difference in the occurrence of adverse events between on-label and off-label prescriptions [12, 28].

Few studies have been conducted in China regarding trends in antipsychotic drug use. During the time period from 1999 to 2008, a report indicated a decrease in the utilization of FGAs and an increase in the use of SGAs in Beijing [29]. According to a study performed in mainland China, the utilization of clozapine decreased from 2002 to 2012 [30]. From 1997 to 2005, an increasing antipsychotic usage trend in the pediatric population was reported in a study conducted in Taiwan, China [31]. In Hong Kong, China, antipsychotic prescriptions for children and adolescents were also evaluated from 2004 to 2014 [32]. However, to the best of our knowledge, research related to antipsychotic prescription trends in pediatric outpatients has not been reported in mainland China.

In this retrospective prescription analysis, we described (i) the trends in the use of antipsychotics and off-label antipsychotics from 2016 to 2021; (ii) the factors associated with the off-label use of antipsychotics; (iii) the trends in different categories of mental illnesses based on The International Statistical Classification of Diseases and Related Health Problems 10th Revision (ICD-10); (iv) antipsychotic polypharmacy in children and adolescents from 2016 to 2021; and (v) drugs combined with antipsychotics.

## Methods

### Population

The participants in our study were recruited from the Hospital Prescription Analysis Cooperative Project. Data on patients from tertiary hospitals in Beijing, Shanghai, Guangzhou, Chengdu, Zhengzhou, Tianjin, Hangzhou, Harbin, and Shenyang were collected through random sampling over 10 workdays for each quarter from 2016 to 2021. The collected information included age, sex, region, department visited, reimbursement type, medications, drug expense, and diagnosis. The inclusion criteria in our study were (i) prescriptions containing at least one antipsychotic agent from 2016 to 2021 and (ii) prescriptions of patients aged 1 to 17 years. The exclusion criteria were (i) repeated prescriptions and (ii) prescriptions without diagnostic information.

### Definition of off-label prescriptions

There are variations in the approved indications for pediatric populations across different countries’ marketing authorizations, which may influence the judgement of off-label prescriptions. In our study, off-label prescriptions were defined as those that did not align with any approved indications by the National Medical Products Administration (NMPA). We also compared the differences in indications between NMPA and the Food and Drug Administration (FDA) (Supplementary Table 1). In this study, we solely analyzed the off-label indications. We did not assess off-label drug dosages due to inadequate information regarding drug dosages. Furthermore, we did not evaluate off-label population usage because certain antipsychotic medication labels do not explicitly specify the precise age range for use in children and adolescents. For example, tiapride was approved for treating alcoholism in children and adolescents, with the conditions of “use with caution” and “decreased dosage”. For indication, the prescriptions for olanzapine, quetiapine, and risperidone were classified as on-label usage for bipolar disorders, despite the lack of specification regarding the types of bipolar disorders. In addition, prescriptions for mental disorders were classified as off-label usage due to the ambiguous nature of the diagnosis.

In addition to analyzing the trends of each prevalent antipsychotic agent, we also explored the most frequent diagnoses associated with antipsychotics and trends for various categories of mental illness. The diagnoses were categorized into nine groups according to the ICD-10: (i) schizophrenia, schizotypal disorders and delusional disorders; (ii) mood or affective disorders; (iii) neurotic, stress-related and somatosensory disorders; (iv) mental developmental disorders; (v) behavioral and emotional disorders, with onset usually occurring in childhood and adolescence; (vi) other mental disorders; (vii) diseases of the nervous system; (viii) diseases of the respiratory system; and (ix) other diseases. In the medication quantity section, we outlined the most prevalent polypharmacy proposals and their respective uses. Moreover, we also included antipsychotic monotherapy in this analysis. Additionally, we listed the drugs that are frequently combined with antipsychotics.

### Statistical analysis

Descriptive statistics were used in our study. For categorical variables, numbers with percentages and chi-square tests were performed. Medians (interquartile ranges [IQRs]) were utilized to describe continuous variables and were compared using the Wilcoxon rank-sum test. To elucidate the factors linked to off-label prescriptions, a multivariate logistic regression analysis was utilized and adjusted for variables deemed to be relevant to off-label usage, including age, sex, years, department visited, reimbursement type, drug expense, antipsychotic type, number of diagnoses, and polypharmacy. The odds ratios (ORs) and 95% confidence intervals (95% CIs) are shown. A p value < 0.05 was considered to indicate statistical significance. All the statistical analyses were performed with R software version 4.2.2 (https://www.r-project.org/). Some graphics were analyzed by using GraphPad-Prism 8.0.

## Results

### Demographic characteristics of the population

**Fig. 1:**
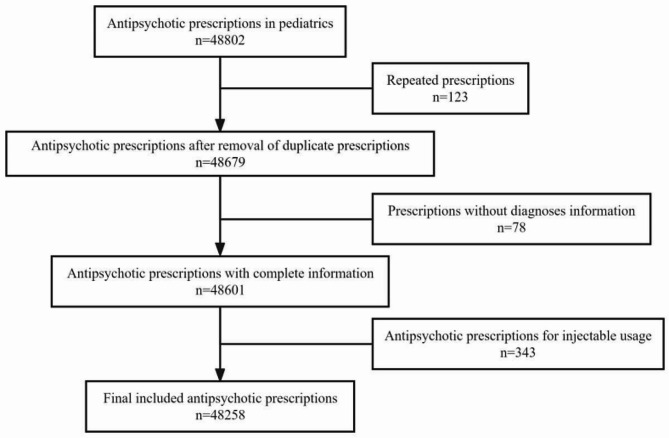
Flow diagram of study inclusion and exclusion criteria

A total of 48,258 prescriptions were extracted from the database based on the inclusion and exclusion criteria (Fig. 1). Among them, 29,813 (61.7%) were off-label. Among the selected children and adolescents, 25,295 (52.4%) were male, and most of the participants were aged 13 to 17 years (63.7%). Most of the patients originated from first-line cities (70.2%), and most of the outpatients were from psychiatric wards (43.7%). With regard to antipsychotics, the proportion of SGAs was greater than that of FGAs (69.6% vs. 28.4%, respectively). In addition, all of the variables were significantly different between on-label and off-label prescriptions (Table 1).

**Table 1:** Demographic characteristics of the population

	Level	Overall (*n* = 48,258)	On-label (*n* = 18,445)	Off-label (*n* = 29,813)	*P* value
Region (%)	First	33,891 (70.2)^a^	11,073 (60.0)	22,818 (76.5)	***< 0.001***
	Non-first	14,367 (29.8)	7372 (40.0)	6995 (23.5)	
Year (%)	2016	6008 (12.4)	2553 (13.8)	3455 (11.6)	***< 0.001***
	2017	6583 (13.6)	2926 (15.9)	3657 (12.3)	
	2018	7299 (15.1)	3490 (18.9)	3809 (12.8)	
	2019	9440 (19.6)	3790 (20.5)	5650 (19.0)	
	2020	8359 (17.3)	2829 (15.3)	5530 (18.5)	
	2021	10,569 (21.9)	2857 (15.5)	7712 (25.9)	
Sex (%)	Male	25,295 (52.4)	11,421 (61.9)	13,874 (46.5)	***< 0.001***
	Female	22,963 (47.6)	7024 (38.1)	15,939 (53.5)	
Age (%)	0–5	4646 (9.6)	1156 (6.3)	3490 (11.7)	***< 0.001***
	6–12	12,850 (26.6)	8051 (43.6)	4799 (16.1)	
	13–17	30,762 (63.7)	9238 (50.1)	21,524 (72.2)	
Department (%)	Psychiatry	21,089 (43.7)	6041 (32.8)	15,048 (50.5)	***< 0.001***
	Neurology	3384 (7.0)	2298 (12.5)	1086 (3.6)	
	Pediatrics	16,366 (33.9)	8112 (44.0)	8254 (27.7)	
	Others	7419 (15.4)	1994 (10.8)	5425 (18.2)	
Reimbursement (%)	Yes	17,712 (36.7)	7478 (40.5)	10,234 (32.3)	***< 0.001***
	No	23,341 (48.4)	8928 (48.4)	14,413 (48.3)	
	Unknown	7205 (14.9)	2039 (11.1)	5166 (17.3)	
Type (%)	Typical	13,702 (28.4)	8845 (48.0)	4857 (16.3)	***< 0.001***
	Atypical	33,593 (69.6)	9017 (48.9)	24,576 (82.4)	
	Typical + Atypical	963 (2.0)	583 (3.2)	380 (1.3)	
Number of polypharmacy (%)	1	24,516 (50.8)	11,908 (64.6)	12,608 (42.3)	***< 0.001***
	2	15,992 (33.1)	4105 (22.3)	11,887 (39.9)	
	3	6177 (12.8)	1833 (9.9)	4344 (14.6)	
	≥ 4	1573 (3.3)	599 (3.2)	974 (3.3)	
Drug expenses (median [IQR])		72.80 [21.00, 231.02]	45.75 [19.00, 250.00]	84.00 [21.84, 215.80]	***< 0.001***
Numbers of diagnoses (%)	1	43,486 (90.1)	16,271 (88.2)	27,215 (91.3)	***< 0.001***
	2	4057 (8.4)	1889 (10.2)	2168 (7.3)	
	≥ 3	715 (1.5)	285 (1.5)	430 (1.4)	

^a^The proportions in parentheses represent the percentage of the specified number within the total count (n) in this column

### Trends in antipsychotic prescription and off-label usage

**Fig. 2:**
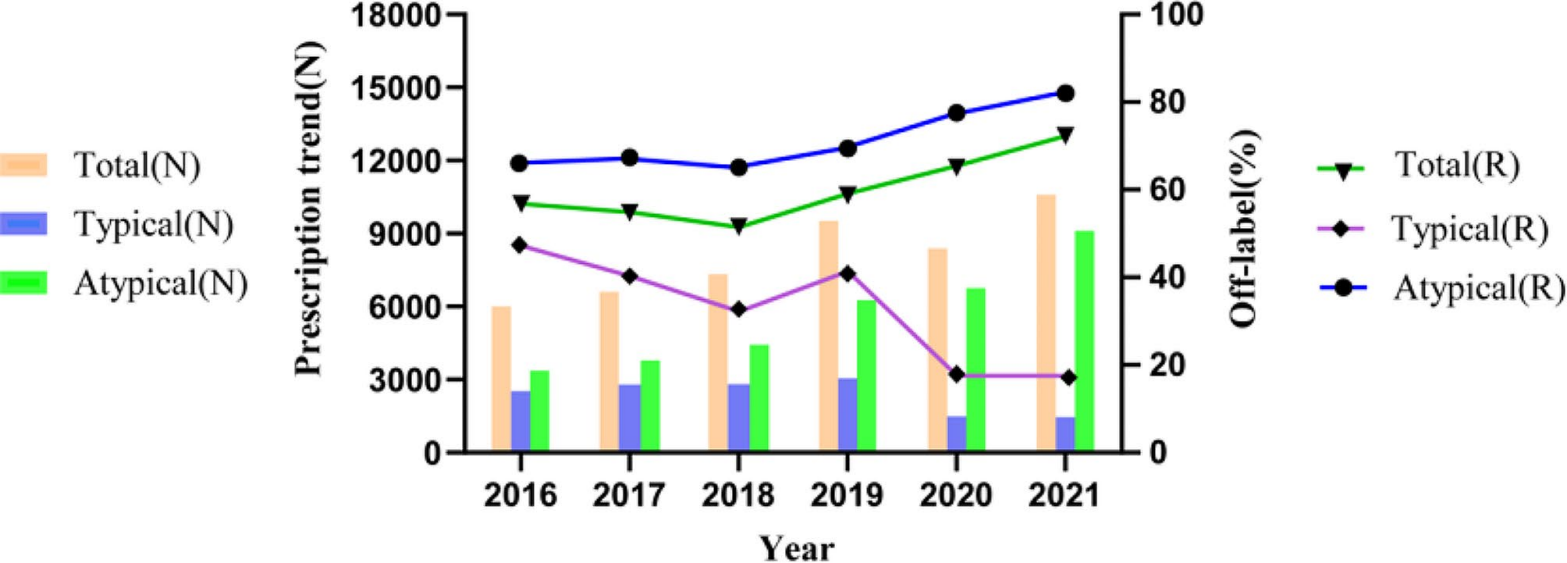
Trends in antipsychotic prescriptions and off-label use.  N: number,  R: rate

As shown in Fig. 2, the proportion of patients receiving antipsychotics generally trended upward from 2016 to 2021. Specifically, the trend of SGAs aligned with the overall trend of total antipsychotic use, whereas the proportion of patients receiving FGAs declined. With respect to the trends in off-label prescriptions, the off-label use of total antipsychotics and SGAs increased from 2016 to 2021, especially for SGAs. Conversely, the proportion of off-label typical antipsychotic prescriptions decreased during this time period.

### Factors associated with off-label prescriptions

The results of multivariate logistic regression analysis showed that all of the variables in our study were associated with off-label prescriptions (Table 2). For instance, the risk in females was greater (OR: 1.07, 95% CI: 1.02–1.12, *p* = 0.005). Compared to that in 2016, the risk in 2018 and 2019 was lower (OR: 0.82, 95% CI: 0.75–0.89, *p* < 0.001; OR: 0.92, 95% CI: 0.86-1.00, *p* = 0.029, respectively), whereas in 2020 and 2021, the risk increased (OR: 1.13, 95% CI: 1.04–1.22, *p* = 0.003; OR: 1.41, 95% CI: 1.30–1.52, *p* < 0.001, respectively). Additionally, the risk in neurology and pediatrics declined compared to that in psychiatry (OR: 0.83, 95% CI: 0.75–0.92, *p* < 0.001; OR: 0.74, 95% CI: 0.70–0.79, *p* < 0.001, respectively). Furthermore, the risk associated with SGAs was much greater than that associated with FGAs (OR: 13.59, 95% CI: 12.54–14.75, *p* < 0.001).

**Table 2:** Multivariable logistic regression analysis for off-label antipsychotic drugs

Variable	OR (95% CI)	*P* value
*Region*		
First-line	–	–
Non-first-line	1.11 (1.05–1.17)	***< 0.001***
*Year*		
2016	–	***–***
2017	0.97 (0.89–1.05)	*0.410*
2018	0.82 (0.75–0.89)	***< 0.001***
2019	0.92 (0.86-1.00)	***0.029***
2020	1.13 (1.04–1.22)	***0.003***
2021	1.41 (1.30–1.52)	***< 0.001***
*Sex*		
Male	–	***–***
Female	1.07 (1.02–1.12)	***0.005***
*Age*		*0.231*
0–5	–	*–*
6–12	0.22 (0.20–0.25)	***< 0.001***
13–17	0.29 (0.26–0.33)	***< 0.001***
*Department*		
Psychiatry	–	***–***
Neurology	0.83 (0.75–0.92)	***< 0.001***
Pediatrics	0.74 (0.70–0.79)	***< 0.001***
Others	2.27 (2.10–2.46)	***< 0.001***
*Reimbursement*		
Yes	–	*–*
No	1.06 (1.01–1.11)	***0.027***
Unknown	4.55 (4.16–4.97)	***< 0.001***
*Type*		
Typical	–	*–*
Atypical	13.59 (12.54–14.75)	***< 0.001***
Typical + atypical	2.28 (1.94–2.67)	***< 0.001***
*Number of polypharmacy*		
1	–	*–*
2	1.57 (1.49–1.66)	***< 0.001***
3	1.29 (1.20–1.39)	***< 0.001***
≥4	0.97 (0.86–1.10)	*0.667*
*Number of diagnoses*		
1	–	*–*
2	0.76 (0.70–0.82)	***< 0.001***
≥3	0.93 (0.77–1.11)	*0.415*
Drug expenses^a^	1.00 (1.00–1.00)	***< 0.001***

^a^The OR and 95% CI for drug expenses were 0.99906 and 0.99900-0.99912, respectively

### Trends for different types of antipsychotics and off-label usage

The results are shown in Table 3. With respect to FGAs, the most frequent antipsychotic used in children and adolescents was tiapride, which was used by 15.8% (*N* = 48,258) of the respondents, followed by chlorpromazine (8.1%) and haloperidol (5.1%). Furthermore, the use of FGAs decreased from 2016 to 2021, with the exception of tiapride, for which the number remained nearly unchanged. For the trends of off-label prescriptions, there was minimal change in the case of chlorpromazine, with over 90% of its prescriptions being off-label. However, the proportion of off-label prescriptions for other FGAs has declined over time. Additionally, the lowest proportion of off-label usage was observed for tiapride, particularly for the treatment of tic disorders. In contrast, the highest rate of off-label prescriptions was found for chlorpromazine, which was mainly used to treat upper respiratory tract infections.

**Table 3:** Trends for different types of antipsychotic prescriptions and off-label usage

Drugs		Total(*n* = 48,258)		2016		2017		2018		2019		2020		2021		Common diagnoses
		N1 (%)^a^	Off-label N2 (%)^b^	N1(%)	Off-label N2 (%)	N1 (%)	Off-label N2 (%)	N1(%)	off-label N2(%)	N1(%)	off-label N2(%)	N1(%)	off-label N2(%)	N1(%)	off-label N2(%)	
Typical	Haloperidol	2444(5.1)	373(15.3)	490(1.0)	82(16.7)	488(1.0)	88(18.0)	477(0.99)	88(18.5)	464(0.96)	63(13.6)	265(0.55)	20(7.5)	260(0.54)	32(12.3)	Tic disorder, epilepsy, schizophrenia
	Sulpiride	962(2.0)	625(65.0)	179(0.37)	129(72.1)	191(0.40)	125(65.3)	241(0.50)	154(63.9)	311(0.64)	203(65.4)	15(0.031)	11(73.3)	25(0.052)	3(12.0)	Mood disorder, schizophrenia, depressive state
	Tiapride	7631(15.8)	519(6.8)	1062(2.2)	76(7.2)	1336(2.8)	109(8.2)	1636(3.4)	127(7.8)	1498(3.1)	83(5.5)	1068(2.2)	57(5.3)	1031(2.1)	67(6.5)	Tic disorder
	Chlorpromazine	3907(8.1)	3774(96.6)	992(2.1)	971(97.9)	859(1.8)	820(95.5)	628(1.3)	605(96.3)	1034(2.1)	1005(97.2)	221(0.46)	212(95.9)	173(0.36)	161(93.1)	Upper respiratory tract infection, autism, epilepsy
Atypical	Aripiprazole	8965(18.6)	7317(81.6)	923(1.9)	717(77.7)	1055(2.2)	804(76.2)	1242(2.6)	909(73.2)	1553(3.2)	1219(78.5)	1761(3.6)	1507(85.6)	2431(5.0)	2161(88.9)	Schizophrenia, depressive state, Tic disorder, mental disorders
	Amisulpride	901(1.9)	525(58.3)	64(0.13)	23(35.9)	123(0.25)	59(48.0)	134(0.28)	68(50.7)	178(0.37)	98(55.1)	173(0.36)	114(65.9)	229(0.47)	163(71.2)	Schizophrenia, mental disorders
	Olanzapine	8787(18.2)	6229(70.9)	1029(2.1)	693(67.3)	1182(2.4)	823(69.6)	1369(2.8)	867(63.3)	1836(3.8)	1205(65.6)	1627(3.4)	1233(75.8)	1744(3.6)	1408(80.7)	Schizophrenia、depressive state, mental disorders
	Quetiapine	8952(18.6)	6442(72.0)	475(0.98)	268(56.4)	535(1.1)	321(60.0)	817(1.7)	535(65.5)	1583(3.3)	1131(71.4)	2213(4.6)	1591(71.9)	3329(6.9)	2596(78.0)	Depressive state, bipolar disorder, Mood disorder
	Risperidone	8346(17.3)	4634(55.5)	1107(2.3)	548(49.5)	1232(2.6)	602(48.9)	1315(2.7)	636(48.4)	1659(3.4)	830(50.0)	1374(2.8)	858(62.4)	1659(3.4)	1160(69.9)	Schizophrenia、mental disorders, autism
	Clozapine	1095(2.3)	549(50.1)	213(0.44)	132(62.0)	161(0.33)	73(45.3)	160(0.33)	71(44.3)	172(0.36)	81(47.0)	162(0.34)	76(46.9)	227(0.47)	116(51.1)	Schizophrenia, mental disorders, bipolar disorder
	Paliperidone	1252(2.6)	876(69.9)	112(0.26)	78(69.6)	149(0.31)	94(63.0)	155(0.32)	75(48.3)	181(0.38)	118(65.1)	273(0.57)	203(74.3)	382(0.79)	308(80.6)	Schizophrenia, mental disorders, bipolar disorder

^a^In the column with N1, the proportions in parentheses represent the percentage of N1 out of the total count (*n* = 48,258); (b) In the column with N2, the proportions in parentheses indicate the percentage of N2 out of the corresponding N1:N_2_/N_1_ × 100%

In terms of SGAs, the most commonly prescribed antipsychotics were aripiprazole (18.6%), quetiapine (18.6%), olanzapine (18.2%), and risperidone (17.3%). Between 2016 and 2021, the use of SGAs generally increased. In reference to off-label usage, the most frequent antipsychotics were aripiprazole (81.6%), quetiapine (72.0%) and olanzapine (70.9%). The most frequently encountered off-label diagnosis was a depressive state. In addition, the patterns for aripiprazole, olanzapine, risperidone, quetiapine, and amisulpride showed overall improvement, whereas the trend for paliperidone initially decreased before increasing again. For clozapine, the off-label usage initially increased but later declined. Additionally, there was minimal variation in the proportions of off-label usage for each SGAs. Furthermore, the majority of SGAs were prescribed for the treatment of schizophrenia.

### Trends in mental illness

The most common disorders in our study were mood or affective disorders (F30-F39, 31.7%) and behavioral and emotional disorders, with onset usually occurring in childhood and adolescence (F90-F98, 29.1%). Specifically, the most frequently diagnosed condition was tic disorder (F95, 22.0%). With respect to the use of antipsychotic medications, there has been an increasing trend in the prevalence of mood or affective disorders; neurotic, stress-related and somatoform disorders; and behavioral and emotional disorders, the onset of which usually occurs in childhood and adolescence. However, for schizophrenia, schizotypal and delusional disorders, as well as for mental developmental disorders, the trends initially increased and then declined. Since 2020, there has been a decline in the use of antipsychotics for nonmental illnesses such as diseases of the nervous system and respiratory system. With respect to off-label usage, the trends generally increased for schizophrenia, schizotypal and delusional disorders, mood or affective disorders, mental developmental disorders, and behavioral and emotional disorders, with onset usually occurring in childhood and adolescence. However, the proportion of off-label antipsychotic prescriptions decreased after 2020 for respiratory system diseases (Table 4).

**Table 4:** Trends for different categories of mental illness based on the ICD-10

Types of diseases	ICD-10	Total(*n* = 48,258)		2016		2017		2018		2019		2020		2021	
		N1(%)^a^	off-label N2(%)^b^	N1(%)	off-label N2(%)	N1(%)	off-label N2(%)	N1(%)	off-label N2(%)	N1(%)	off-label N2(%)	N1(%)	off-label N2(%)	N1(%)	off-label N2(%)
Schizophrenia, schizotypal and delusional disorders	F20–F29	4855(10.1)	929(19.1)	679(1.4)	106(15.6)	842(1.7)	165(19.6)	888(1.8)	102(11.5)	943(2.0)	154(16.3)	764(1.6)	194(25.4)	739(1.5)	208(28.1)
Mood [affective] disorders	F30–F39	15,280(31.7)	11,340(74.2)	861(1.8)	499(58.0)	1073(2.2)	684(63.7)	1642(3.4)	1073(65.3)	2896(6.0)	2064(71.3)	3580(7.4)	2742(76.6)	5228(10.8)	4278(81.8)
Neurotic, stress-related and somatoform disorders	F40,–F48	4758(9.9)	4298(90.3)	519(1.1)	419(94.4)	573(1.2)	524(91.4)	641(1.3)	559(87.2)	954(2.0)	812(85.1)	901(1.9)	828(91.9)	1170(2.4)	1085(92.7)
Mental developmental disorder	F70–F89	2444(5.1)	987(40.4)	344(0.71)	120(34.9)	404(0.84)	150(37.1)	415(0.86)	157(37.8)	532(1.1)	173(32.5)	371(0.77)	196(52.8)	378(0.78)	191(50.5)
Behavioral and emotional disorders with onset usually occurring in childhood and adolescence	F90–F98	14,029(29.1)	5032(35.9)	1929(4.0)	549(28.5)	2197(4.6)	623(28.4)	2596(5.4)	723(27.9)	2642(5.5)	889(33.6)	2161(4.5)	927(42.9)	2504(5.2)	1321(52.8)
Other mental disorders	F99, F06, F07, F09,F50,F51	3713(7.7)	3511(94.6)	604(1.3)	569(94.2)	579(1.2)	535(92.4)	525(1.1)	481(91.6)	580(1.2)	542(93.4)	625(1.3)	600(96.0)	800(1.7)	784(98.0)
Diseases of the nervous system	G00–G99	1563(3.2)	1333(85.3)	242(0.50)	214(88.4)	293(0.61)	268(91.5)	264(0.55)	234(88.6)	337(0.70)	277(82.2)	204(0.42)	161(78.9)	223(0.46)	179(80.3)
Diseases of the respiratory system	J00–J99	3838(8.0)	3393(88.4)	995(2.1)	923(92.8)	808(1.7)	716(88.6)	618(1.3)	523(84.6)	995(2.1)	910(91.5)	206(0.43)	152(73.8)	216(0.45)	169(78.2)
Other diseases	A49, C30, E34, E55, E60, E83, K29, K30, K31, K52, K59, M30, M32, Q21	740(1.5)	253(34.2)	83(0.17)	36(43.4)	108(0.22)	53(49.1)	131(0.27)	29(22.1)	187(0.39)	53(28.3)	109(0.23)	40(36.7)	122(0.25)	42(34.4)

^a^In the column with N1, the proportions in parentheses represent the percentage of N1 out of the total count (*n* = 48,258); (b) In the column with N2, the proportions in parentheses indicate the percentage of N2 out of the corresponding N1:N_2_/N_1_ × 100%

### Antipsychotic medication quantity

The most common antipsychotic pattern was monotherapy with antipsychotics (50.8%), followed by combination therapy of antipsychotics with another drug (33.1%). Among the single-drug regimens, tiapride (28.3%), chlorpromazine (14.6%), risperidone (14.5%), aripiprazole (13.5%), olanzapine (9.1%), and quetiapine (7.8%) were frequently prescribed for the treatment of tic disorders, upper respiratory tract infections, and schizophrenia. In polypharmacy protocols involving two drugs, the most common combination was antipsychotics with sertraline, and the most prevalent diagnosis associated with this combination was a depressive state. Additionally, the common treatment regimen often involves the use of two antipsychotic medications. In combination therapy involving 3 drugs, the most common diagnoses were depressive state, bipolar disorder, and schizophrenia. With regard to treatment regimens involving more than 4 drugs, bipolar disorder was the most common diagnosis (Table 5).

**Table 5:** Antipsychotic medication quantity in children and adolescence

Medication quantity	N1 (%)	Off-label N2 (%)	Common antipsychotic schemes (N3, %)	Common diagnoses
1	24,516(50.8)	12,608 (51.4)	Tiapride (6934, 28.3), Chlorpromazine (3587, 14.6), Risperidone (3555, 14.5), Aripiprazole (3311, 13.5), Olanzapine (2248, 9.1), Quetiapine (1930, 7.8)	Tic disorder, upper respiratory tract infection, schizophrenia, mental disorders, depressive state
2	15,992(33.1)	11,887(74.3)	Quetiapine + Sertraline (1814, 11.3), Aripiprazole + Sertraline (1260, 7.8), Olanzapine + Sertraline(1192, 7.4), Risperidone + Sertraline(821, 5.1), Quetiapine + Lithium(783, 4.8), Olanzapine + Fluoxetine(506, 3.1), Quetiapine + Fluoxetine (504, 3.1), Quetiapine + fluvoxamine (367, 2.2), Aripiprazole + Olanzapine (352, 2.2), Aripiprazole + fluvoxamine (336, 2.1)	Depressive state, schizophrenia, bipolar disorder, mental disorders, tic disorder
3	6177(12.7)	4144(70.3)	Quetiapine + Sertraline + Lithium(207, 3.4), Olanzapine + Sertraline + Lithium(127, 2.1), Quetiapine + Alprazolam + Sertraline (123, 2.0), Aripiprazole + Olanzapine + Sertraline(110, 1.8), Olanzapine + Alprazolam + Sertraline(101, 1.6)	Depressive state, bipolar disorder, schizophrenia, mental disorders, mood disorder
≥ 4	1573(3.2)	974(61.9)	Olanzapine + Sulpiride + Flupentixol/Melitracen + Fluvoxamine(22, 1.4), Aripiprazole + Risperidone + Sertraline + Lithium(18, 1.1), Quetiapine + Alprazolam + Sertraline + Lithium (17, 1.1), Olanzapine + Buspirone + Sertraline + Lithium (16, 1.0), Olanzapine + Lorazepam + Sertraline + Lithium (15, 1.0)	Bipolar disorder, depressive state, mood disorder, schizophrenia, mental disorders

In the column with N1, the proportions in parentheses represent the percentage of N1 out of the total count (*n* = 48,258); b. In the column with N2, the proportions in parentheses indicate the percentage of N2 out of the corresponding N1:N_2_/N_1_ × 100%; c. Within columns featuring N3, the proportions enclosed in parentheses denote the proportion of N3 in the corresponding N1:N_3_/N_1_ × 100%

Among nonantipsychotic drugs, antidepressants, such as sertraline (*N* = 8,226, 17.0%), fluvoxamine (*N* = 2,379, 4.9%), and fluoxetine (*N* = 2,267, 4.7%), were the most frequently combined with antipsychotics. Another frequently prescribed medication was lithium (*N* = 3,943, 8.2%), which is classified as being an antimanic drug. In addition to antidepressants and antimanic medications, antipsychotics were also coprescribed with anxiolytics, sedative-hypnotics, and psychostimulants (Supplementary Table 2).

## Discussion

In this retrospective study, we analyzed the trends in antipsychotic and off-label usage in children and adolescents from 2016 to 2021. To the best of our knowledge, this was the first study related to trends in the use of antipsychotics and off-label prescriptions in children and adolescents in China. Our study assessed the real-world usage of antipsychotics, which provides insights into how these drugs are actually being used in clinical practice.

In our study, we noted that the safety and efficacy of certain antipsychotics in pediatric patients have not been established, and several antipsychotics were not authorized for use in the pediatric population. Moreover, information regarding drug dosage was incomplete. As a result, our study focused solely on analyzing off-label indications. In addition, we discovered that certain antipsychotics, such as sulpiride and tiapride, were not included in the FDA list. Furthermore, the indications approved by the NMPA for antipsychotics vary from those approved by the FDA. For instance, in China, aripiprazole is solely approved for treating schizophrenia, whereas in addition to this indication, it has also been approved for bipolar I disorder, irritability associated with autistic disorder, and Tourette’s disorder. Consequently, off-label prescriptions were assessed based on the NMPA, which aligns more closely with the circumstances in China. Furthermore, the results of our study may differ from those of studies conducted in other countries.

Our study demonstrated a general increase in both the prescription of antipsychotics and their off-label usage between 2016 and 2021. Notably, the number of patients receiving antipsychotics decreased in 2020, which may be related to the prevention and control policy for COVID-19 at that time [33]. In addition, we noticed a decline in the trends of typical antipsychotic prescriptions and off-label usage from 2016 to 2021, whereas the proportions of SGAs increased, thus mirroring the findings in other studies [31, 32, 34, 35]. For example, the prevalence of SGAs increased from 1997 to 2005 and from 2004 to 2014 in studies conducted in Taiwan [31] and Hong Kong [32], China.

Furthermore, we observed that 61.7% of the usage was off-label, which is consistent with findings from previous studies [12, 32, 36]. Due to the vague definition of a suitable population of children and adolescents approved by the NMPA or FDA, we focused solely on off-label indications in our analysis. As a result, the proportion of off-label usage may be lower than the actual situation. Moreover, prescriptions for treating bipolar disorders with medications such as olanzapine, quetiapine, and risperidone were classified as on-label usage. This contributed to the lower rate of off-label usage.

In our study, age, sex, region, years, department, antipsychotic type, drug expenses, polypharmacy frequency and number of diagnoses were found to be associated with off-label prescriptions, which was essentially consistent with the findings of other studies [37–40]. However, the risks for off-label prescriptions of female sex and SGAs were greater in our study, which was different from the results obtained in the Netherlands [40] and United States [39]. Additionally, we observed that the risk of off-label usage declined in 2018 and 2019 and then increased in 2020 and 2021, which may be related to the COVID-19. Studies have shown that antipsychotic prescriptions increased after the outbreak of COVID-19 [33, 41, 42]. However, there is no direct evidence showing that increasing off-label antipsychotic prescriptions are associated with COVID-19, and additional studies on this topic are needed.

The highest proportion of off-label prescriptions exceeded 90% for chlorpromazine. This was primarily due to its utilization in treating upper respiratory tract infections in children and adolescents. Chlorpromazine was approved for the treatment of schizophrenia, nausea and vomiting instead of upper respiratory tract infections. However, as one of the components of the lytic cocktail, chlorpromazine has been widely used for preoperative sedation since its introduction [43, 44]. In addition, chlorpromazine has been shown to cause cooling and respiratory inhibition [45, 46]. These findings may contribute to the widespread usage of chlorpromazine in children and adolescents.

For SGAs, the most frequent off-label antipsychotic was aripiprazole, followed by quetiapine and olanzapine. Aripiprazole has been approved by the NMPA solely for the treatment of schizophrenia. Correspondingly, FDA approval included additional indications, such as the treatment of bipolar disorder, irritability associated with autistic disorder, and Tourette’s disorder. Concerning olanzapine, the FDA has approved its use for treating resistant depression, whereas the NMPA has not granted approval for this indication. With regard to off-label use for treating depressive states, some RCTs have shown that aripiprazole and quetiapine could be used as adjunctive treatments for major depressive disorder [47–52]. These findings may be related to the off-label use of aripiprazole, quetiapine and olanzapine.

The increase in SGAs from 2016 to 2021 may be related to the increase in mood or affective disorders and neurotic, stress-related and somatoform disorders in children and adolescents. For these diseases, the rates of off-label prescriptions were greater than those for other diseases and increased over the six years. This may be responsible for the increasing proportions of off-label usage in SGAs. Except for schizophrenia, the most common diagnoses were depressive state and mental disorders, which were classified as off-label usage in SGAs, thus causing a higher rate of off-label prescriptions compared to FGAs.

One of the most common diagnoses in our study was behavioral and emotional disorders, with onset usually occurring in childhood and adolescence (F90-F98), which was consistent with the results of other studies [9, 21, 31, 53]. In accordance with a study conducted in Taiwan [31], the most common diagnosis in pediatric patients in our study was tic disorder (F95, 22.0%). However, studies conducted in the United States and Germany indicated that the most common diagnosis in children and adolescents who received antipsychotics was hyperkinetic syndrome of childhood (F90) [9, 12, 39, 53]. Additionally, our study revealed a high prevalence of mood or affective disorders among pediatric patients. Furthermore, we observed a significant increase in the prescription rates of antipsychotics, including those for off-label use, for treating mood or affective disorders in this population between 2016 and 2021. These findings underscore the need for prioritizing psychological issues in children and adolescents and advocating for evidence-based and standardized approaches to antipsychotic usage in pediatric patients.

In our study, we observed that antipsychotic monotherapy was of high frequency, constituting more than 50%. For the schemes containing two drugs, antipsychotics were often combined with antidepressants for the treatment of depressive states, thus causing the highest risk in polypharmacy with two drugs compared to other polypharmacy. Additionally, polypharmacy with two or more antipsychotics is common in children and adolescents. This may lead to greater harm due to the increased incidence of adverse reactions compared to antipsychotic monotherapy. According to guidelines [6, 54, 55], the primary choice is to choose monotherapy for treatment, and clozapine or polypharmacy with antipsychotics are recommended when monotherapy was ineffective. However, evidence-based polypharmacy is needed. Currently, there is insufficient evidence to support the superiority of antipsychotic polypharmacy over antipsychotic monotherapy in terms of effectiveness [56].

In our study, we observed that patients with common off-label usage and polypharmacy of antipsychotics were mostly diagnosed with a depressive state. Studies have suggested the efficacy of certain antipsychotics as adjunctive treatments for depressive disorder [48, 50]. Meanwhile, certain antipsychotics have received approval for treating depressive symptoms, such as sulpiride. These may lead to the widespread use of antipsychotics in depressive pediatrics. Furthermore, it is worth noting that depressive state was not the same as depressive disorder. It is widely recognized that during both the acute and remission phases of schizophrenia, individuals may experience depressive symptoms. Additionally, among individuals diagnosed with bipolar disorder, it was common for them to experience periods marked by depressive symptoms. Therefore, depressive state may represent depressive symptoms during episodes of schizophrenia and bipolar disorder. This may also be responsible for the use of antipsychotics in pediatric patients with depressive states.

In alignment with depressive symptoms, antidepressants emerged as the most frequently prescribed medication in association with antipsychotics. Among these medications, sertraline was identified as being the most frequent drug for children and adolescents. Similar to fluvoxamine and fluoxetine, sertraline has been approved for the treatment of depression. However, none of these three drugs were authorized for depression treatment in children and adolescents, except for fluoxetine, which obtained FDA approval for major depressive disorder in the pediatric population over the age of 8 years. Concerning another frequently prescribed non-antipsychotic drug in our study, lithium has received approval for the treatment of mania, bipolar disorder, schizoaffective disorder, and recurrent major depressive disorder in children and adolescents over the age of 12 years. It was also authorized for bipolar disorder in the pediatric population over 7 years of age according to FDA indications. Given the high prevalence of mood or affective disorders (F30-F39), these medications were commonly prescribed.

There were several limitations in our study. First, the prescriptions were extracted from outpatients instead of from hospitalized patients. As a result, the findings of our study may not reflect all the situation of the entire pediatric population. Nevertheless, given the larger number of outpatients compared to inpatients, which provides a more substantial reference for medical professionals and the public, we believe that the results from outpatients may better reflect the pediatric population as a whole than those from inpatients. Meanwhile, other studies related to antipsychotic medications also use outpatients as their research population [9, 21, 57]. Second, certain diagnostic information within the database lacked standardization. For instance, certain prescriptions associated with bipolar disorder diagnoses did not provide specific details regarding the type of bipolar disorder, thus posing challenges in determining whether the use was on-label or off-label. In our study, these prescriptions were identified as on-label prescriptions if bipolar disorder patients were treated with olanzapine, quetiapine, or risperidone. Finally, in terms of factors associated with off-label prescriptions, some unknown factors may not have been included in the multivariate logistic regression analysis. Therefore, the unknown confounding factors could not be adequately adjusted for in our analysis.

## Conclusion

In our study, we found that antipsychotic prescriptions among children and adolescents increased from 2016 to 2021. Furthermore, off-label antipsychotic usage has also increased. Specifically, atypical antipsychotic prescriptions increased, whereas typical antipsychotic prescriptions declined. In addition, factors such as non-first-line areas, female, and atypical antipsychotics were identified as risk factors associated with off-label usage. In conclusion, the off-label use of antipsychotics is common in the pediatric population; however, more studies and evidence-based support are needed.

### Electronic supplementary material

Below is the link to the electronic supplementary material.

Supplementary Material 1

## Data Availability

The data supporting the results in our study are available from the corresponding author with a reasonable request.
