# Supplementary material for: Trends and off-label utilization of antipsychotics in children and adolescents from 2016 to 2021 in China: a real-world study
Source: Child Adolesc Psychiatry Ment Health. 2024 Jun 21;18:77. doi: 10.1186/s13034-024-00766-4 (PMC11193198; doi:10.1186/s13034-024-00766-4)
Supplement: Supplementary file 1 — Supplementary Material 1 [file 13034_2024_766_MOESM1_ESM.docx]

# Trends and off-label utilization of antipsychotics in children and adolescents from 2016 to 2021 in China: a real-world study

Wang Zhaojian2,4#, Jiang Meizhu1,4#, Hong Jun3,4, Guo Shanshan1, Huo Jiping1, Zhao Zhigang1, Gong Ying5*, Li Cao1*

1. Department of Pharmacy, Beijing Tiantan Hospital, Capital Medical University, Beijing, China

2. Department of Pharmacy, Beijing Shijitan Hospital, Capital Medical University, Beijing, China

3. Department of Pharmacy, Beijing Chao-Yang Hospital, Capital Medical University, Beijing, China

4. Department of Clinical Pharmacology, School of Pharmaceutical Sciences, Capital Medical University, Beijing, China

5. Department of Pharmacy, Dongfang Hospital, Beijing University of Chinese Medicine, Beijing, China

^#^ These authors contributed equally to this article

*Corresponding Authors:

Ying Gong, [dfyygong@126.com](mailto:dfyygong@126.com), department of Pharmacy, Dongfang Hospital, Beijing University of Chinese Medicine, Beijing, China, No. 6, Phase 1, Fangxingyuan, Fangzhuang, Fengtai District, Beijing, China.

Cao Li, [licao@bjtth.org](mailto:licao@bjtth.org), department of Pharmacy, Beijing Tiantan Hospital, Capital Medical University, Beijing, 100050, China, 119 Nan Si Huan Xi Lu, Fengtai District, Beijing, China.

Table 1. Approved indication and population in NMPA and FDA

| Type | Drug | NMPA | | FDA | |
| --- | --- | --- | --- | --- | --- |
|  |  | Indication | Age | Indication | Age |
| Typical | Haloperidol | Schizophrenia | Over 3, use with caution and discretionary decrement | Schizophrenia | Over 3 |
|  |  | Tourette's Disorder |  | Tourette's Disorder | Over 3 |
|  |  | Organic mental disorders and senile mental disorders |  | Hyperactive behavior | Over 3 |
|  |  | Autism |  |  |  |
|  |  | Mania |  |  |  |
|  |  | Aggressive behavior，agitation and antagonism |  |  |  |
|  | Droperidol | Schizophrenia | Use with caution | Nausea/Vomiting | Over 2 |
|  |  | Combined with fentanyl for anesthesia |  |  |  |
|  |  | Mania |  |  |  |
|  | Sulpiride | Schizophrenia | Over 6 | Unlisted |  |
|  |  | Depressive symptom |  |  |  |
|  |  | Vomiting |  |  |  |
|  | Tiapride | Tourette's Disorder | 7-12 | Unlisted |  |
|  |  | Alcoholism | use with caution and discretionary decrement |  |  |
|  |  | Chorea |  |  |  |
|  |  | Headache, neuromuscular pain, cramp |  |  |  |
|  | Chlorpromazine | Schizophrenia | Younger than 6: use with caution; over 6: discretionary decrement | Schizophrenia | Over 6 months |
|  |  | Severe nausea and vomiting |  | Nausea, hiccups and vomiting | Over 6 months |
|  |  |  |  | Preoperative sedation | Over 6 months |
|  |  | Mania |  | Bipolar disorder, mania | 1-12 |
|  |  | Hallucinations and delusions |  | Severe behavioral problems in children | 1-12 |
|  |  |  |  | Acute intermittent porphyria | Not mentioned in pediatrics |
|  |  |  |  | Tetanus (adjunct) | Not mentioned in pediatrics |
|  | Penfluridol | Schizophrenia | Discretionary decrement | Unlisted |  |
|  |  | Hallucinations, delusions, Withdrawal, apathy and symptoms |  |  |  |
|  | Flupentixol | Combined with Melitracen for schizophrenia, anxiety, depression and neurasthenia | Not recommended in pediatrics | Unlisted |  |
|  | Perphenazine | Schizophrenia | ≥12 | Schizophrenia | ≥12 |
|  |  | Severe nausea and vomiting |  | Severe nausea and vomiting |  |
|  |  | Mania |  |  |  |
|  |  | Stupor |  |  |  |
|  |  | Hallucinations and delusions |  |  |  |
|  |  | Anxiety symptoms |  |  |  |
| Atypical | Aripiprazole | Schizophrenia | 13-17 | Schizophrenia | 13-17 |
|  |  |  |  | Bipolar I Disorder, manic or mixed episodes | 10-17 |
|  |  |  |  | Irritability associated with autistic disorder | 6-17 |
|  |  |  |  | Tourette’s Disorder | 6-17 |
|  | Amisulpride | Schizophrenia | 15-18: Not recommended | Postoperative nausea and vomiting（PONV） | Safety and effectiveness in pediatric patients have not been established |
|  | Olanzapine | Schizophrenia | Not recommended in pediatrics | Schizophrenia | 13-17 |
|  |  | Moderate to severe manic episode |  | Bipolar I disorder, manic or mixed episodes | 13-17 |
|  |  | Relapse of bipolar disorder（mania） |  | Combined witn Flouxetine for Depressive Episodes associated with Bipolar I Disorder | 10-17 |
|  |  |  |  | Resistant depression | Adults |
|  | Quetiapine | Schizophrenia | Not recommended in pediatrics | Schizophrenia | 13-17 |
|  |  | Bipolar disorder, manic or depressive episode |  | Bipolar I disorder, manic episodes | 10-17 |
|  | Risperidone | Schizophrenia | 13-17 | Schizophrenia |  |
|  |  | Bipolar I disorder, manic episodes | 10-17 | Bipolar I disorder, manic or mixed episodes | 10-17 |
|  |  | Autism | 5-17 | Irritability associated with autistic disorder | 5-16 |
|  |  | Persistent aggression or other destructive behavior associated with mental retardation or conduct disorder | 5-17 |  |  |
|  | Clozapine | Resistant schizophrenia | ≥12 | Resistant schizophrenia | Safety and effectiveness in pediatric patients have not been established |
|  |  |  |  | Schizophrenia or schizoaffective disorder |  |
|  | Paliperidone | Schizophrenia | 12-17 | Schizophrenia | 12-17 |
|  | Perospirone | Schizophrenia | Not recommended in pediatrics | Unlisted |  |
|  | Ziprasidone | Schizophrenia | Not recommended in pediatrics | Schizophrenia | Safety and effectiveness in pediatric patients have not been established |
|  |  |  |  | Bipolar I disorder, manic or mixed episodes |  |
|  | Bunamselin | Schizophrenia | Not recommended in pediatrics | Unlisted |  |

Supplementary Table 2 Most commonly used drugs in combination with antipsychotic medications

| Drugs | Types | N |
| --- | --- | --- |
| Sertraline | antidepressant | 8226 |
| Lithium | antimanics | 3943 |
| Fluvoxamine | antidepressant | 2379 |
| Fluoxetine | antidepressant | 2267 |
| Alprazolam | anxiolytic; sedative-hypnotics | 1663 |
| Lorazepam | anxiolytic; sedative-hypnotics | 1091 |
| Escitalopram | antidepressant | 940 |
| Tomoxetine | psychostimulant | 858 |
| Oxazepam | anxiolytic; sedative-hypnotics | 715 |
| Duloxetine | antidepressant | 532 |
| Tandospirone | anxiolytic | 475 |
| Venlafaxine | antidepressant | 462 |
| Methylphenidate | psychostimulant | 426 |
| Agomelatine | antidepressant | 338 |
| Huperzine-A | psychostimulant | 333 |
| Buspirone | anxiolytic | 319 |
| Estazolam | sedative-hypnotics | 272 |
| Clonazepam | sedative-hypnotics | 253 |
| Paroxetine | antidepressant | 221 |
| Zolpidem | sedative-hypnotics | 221 |
